# Precisely Engineered Supported Gold Clusters as a Stable Catalyst for Propylene Epoxidation

**DOI:** 10.1002/anie.202104952

**Published:** 2021-07-09

**Authors:** Nidhi Kapil, Tobias Weissenberger, Fabio Cardinale, Panagiotis Trogadas, T. Alexander Nijhuis, Michael M. Nigra, Marc‐Olivier Coppens

**Affiliations:** ^1^ Centre for Nature Inspired Engineering and Department of Chemical Engineering University College London London WC1E 7JE UK; ^2^ SABIC Europe Geleen, Limburg 6167 RD The Netherlands; ^3^ Department of Chemical Engineering University of Utah Salt Lake City UT 84112 USA

**Keywords:** catalyst stability, epoxidation, gold, heterogeneous catalysis, propylene

## Abstract

Designing a stable and selective catalyst with high H_2_ utilisation is of pivotal importance for the direct gas‐phase epoxidation of propylene. This work describes a facile one‐pot methodology to synthesise ligand‐stabilised sub‐nanometre gold clusters immobilised onto a zeolitic support (TS‐1) to engineer a stable Au/TS‐1 catalyst. A non‐thermal O_2_ plasma technique is used for the quick removal of ligands with limited increase in particle size. Compared to untreated Au/TS‐1 catalysts prepared using the deposition precipitation method, the synthesised catalyst exhibits improved catalytic performance, including 10 times longer lifetime (>20 days), increased PO selectivity and hydrogen efficiency in direct gas phase epoxidation. The structure‐stability relationship of the catalyst is illustrated using multiple characterisation techniques, such as XPS, ^31^P MAS NMR, DR‐UV/VIS, HRTEM and TGA. It is hypothesised that the ligands play a guardian role in stabilising the Au particle size, which is vital in this reaction. This strategy is a promising approach towards designing a more stable heterogeneous catalyst.

## Graphical abstract

A stable and selective Au/TS‐1 catalyst is synthesised for direct gas phase epoxidation of propylene to propylene oxide, a key commodity chemical. The synthesis methodology is highly versatile for obtaining a stable, supported gold catalyst. The stabilising ligands play an important role in providing enhanced stability to the Au nanoparticles.

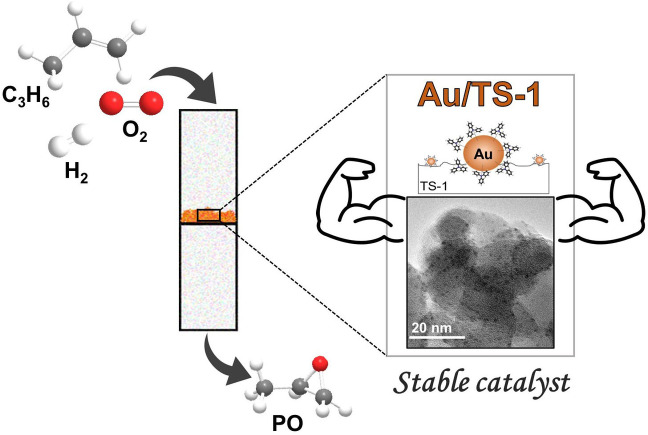

## Introduction

Propylene oxide (PO) is a high value‐added commodity chemical, because it serves as a starting material to synthesise polyether polyols and propene glycol, which are subsequently used to produce polyurethane foams and polyesters, respectively.^[1]^ The current annual PO production is more than 10 million tons world‐wide, and demand is increasing remarkably.^[2]^ However, the conventional methods to produce PO (chlorohydrin and the hydroperoxide process) suffer from major drawbacks, like toxic waste generation, complicated multistep processing, and formation of by‐products in a fixed ratio, which rely heavily on market demands to be profitable.[^1b^, ^3^] DOW‐BASF and EVONIK‐ ThyssenKrupp have independently commercialised the HPPO (hydrogen peroxide to propylene oxide) process, which employs hydrogen peroxide as an oxidant, using TS‐1 as a catalyst, demonstrating the economic feasibility of utilising hydrogen, provided the hydrogen is used efficiently as sacrificial reductant in this hydro‐epoxidation.^[4]^ The HPPO process is a ground‐breaking route to produce propylene oxide from propylene, but the main disadvantage of this method is that it employs multiple reactors, and requires a separate hydrogen peroxide production plant.[^3a^, ^4a^, ^5^]

Ever since Haruta and Hutchings discovered the impressive catalytic properties of gold nanoparticles, the direct gas phase epoxidation of propylene using H_2_ and O_2_ has gained considerable attention as a green, simple and environmentally benign route for PO synthesis.^[6]^ It is known that H_2_ reacts with O_2_ over the surface of highly dispersed gold nanoparticles to generate in situ peroxo species, along with tetrahedrally coordinated Ti^4+^ sites, which enable subsequent epoxidation of propylene to PO.[^6b^, ^6c^, ^7^] A single‐step route is highly desirable to achieve a cost‐effective and environmentally friendly process. Moreover, using H_2_ and O_2_ as reactants is a step towards a renewable route, as both H_2_ and O_2_ can be produced from renewable electricity by electrolysis. Numerous reports of various gold nanoparticles supported on Ti‐containing materials showcase good activity and selectivity; however, they do not exhibit a particularly long catalyst lifetime.^[8]^ A long catalyst lifetime means that the catalytic performance, including activity and selectivity, should be stable over an appreciable time‐on‐stream, or the catalyst should easily be regenerated to a similar performance level. The direct epoxidation of propylene with Au/Ti‐containing catalysts could eventually become an alternative route to other, indirect or multistep production methods to propylene oxide, but two significant impediments are poor hydrogen utilisation and low catalyst stability. There is still a long way towards commercialisation, but this work represents a step towards improving catalyst stability, hydrogen utilisation, and PO selectivity.

Catalyst stability is an important parameter that cannot be neglected, especially in industrial processes. A considerable amount of time and resources are spent on catalyst replacement.^[9]^ This process can cost billions of dollars per year to the industry.^[10]^ The maximum catalyst stability reported for the direct gas phase reaction in literature so far is ca. 250 h in a microreactor setup with periodic regenerations, using Au/TS‐1 catalyst prepared by a deposition precipitation (DP) method.^[5a]^ Thus, low catalyst stability and poor hydrogen efficiency are key issues that still need to be addressed to make the single‐step process using Au‐based catalysts economically viable. The deactivation of these catalysts mainly originates from blocking of active sites by adsorption of oxygenate species or metal particle sintering.[^5a^, ^11^] Although catalyst deactivation is inevitable, it can be delayed or reduced, by carefully designing the catalyst architecture using different synthetic approaches and tuning the metal‐support interaction to impart extra stability.^[12]^ The DP method is commonly employed to deposit gold on supports and is based on the isoelectric point of the support material.^[13]^ This catalyst synthesis strategy also presents some disadvantages, such as nanoparticle aggregation, non‐homogeneous particle size distributions, and weak affinity toward the support, making it a difficult to reproduce technique.[^13^, ^14^] Therefore, designing a stable, yet selective catalyst becomes an area of key importance from both academic and industrial points of view.

Herein, we report a facile one‐pot methodology to synthesise very stable, sub‐nanometre gold clusters (ca. 0.8 nm core size) using triphenylphosphine as a stabilising ligand. These pre‐formed gold clusters can be directly immobilised onto the zeolitic support (in this work, TS‐1) without any notable change in the shape and size of the clusters. We demonstrate a non‐thermal plasma technique as a fast, efficient, and effective treatment for the removal of bound ligands that provides considerable advantages over thermally driven oxidative procedures. The catalytic behaviour of this catalyst and untreated Au/TS‐1 catalysts are subsequently compared in direct propylene epoxidation with H_2_ and O_2_. The catalyst demonstrates a good activity and selectivity over several regeneration cycles; however, longer testing will be required in a pilot scale reactor to assess its total lifetime in the future.

## Results and Discussion

**Figure 1:**
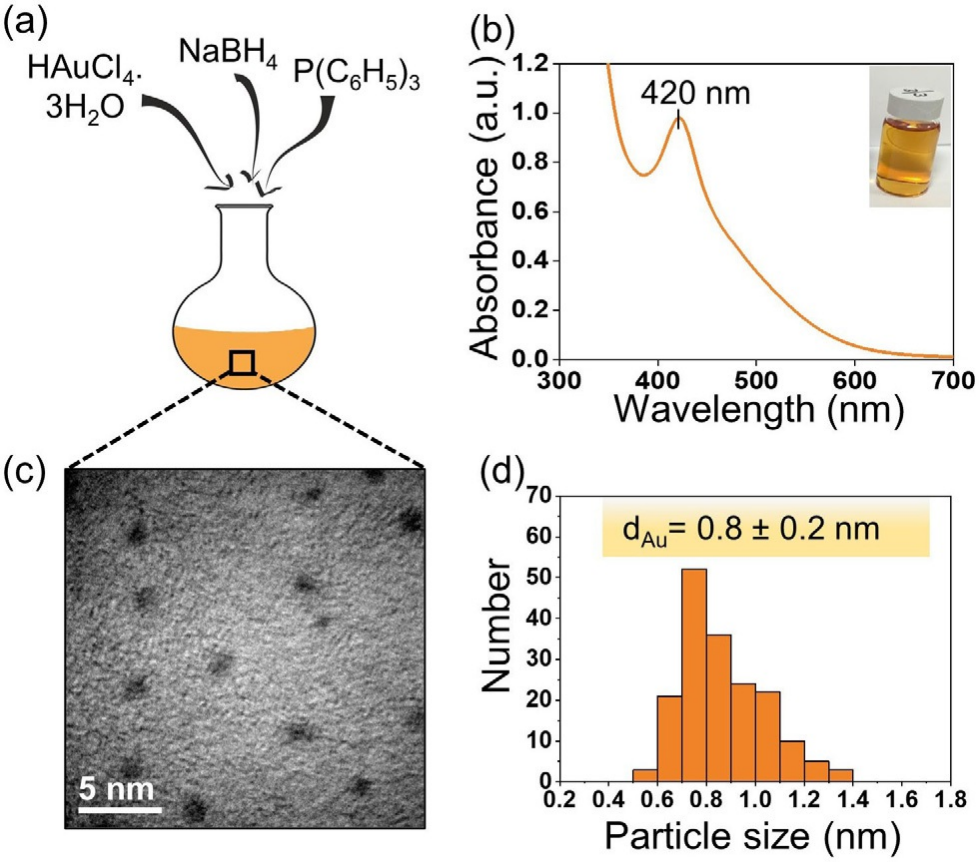
Synthesis and characterisation of Au nanoclusters. a) Illustration of the synthesis of Au nanoclusters using a one‐pot approach. b) UV/Vis spectrum (inset shows vial containing Au clusters in ethanol). c) Transmission electron microscopy image. d) Particle size distribution histogram.

Figure 1 a illustrates the synthesis procedure for Au nanoclusters. These nanoclusters are prepared using gold (III) chloride trihydrate (HAuCl_4_⋅3 H_2_O) as gold precursor. The gold precursor is dissolved in ethanol and stirred for 15 minutes. Afterwards, an ethanolic solution of triphenylphosphine (TPP) is added to the gold solution. TPP ligands are nucleophiles, which possess a lone pair of electrons; they play an important role in this reaction, acting as a reducing agent before helping to stabilise the Au nanocluster intermediate.^[15]^ The gold precursor, which is yellow in colour, changes to colourless after the addition of TPP, due to reduction of Au^III^ to Au^I^, leading to formation of a coordination complex (Cl‐Au^I^‐PPh_3_) with TPP ligands. Subsequently, sodium borohydride (NaBH_4_), a strong reducing agent, is added to the above Au^I^ complex, converting Au^I^ to Au^0^, and resulting in the formation of sub‐nanometre Au clusters stabilised by phosphine ligands. Various concentrations of NaBH_4_ (1, 4, 10 and 20 equiv.) are tested for optimisation and 4 equivalents proves to be the most suitable concentration for the synthesis of the smallest gold clusters, as confirmed by UV/Vis spectroscopy (Supporting Information, Figure S1). Figure S1 shows photographs and UV/Vis spectra of different Au nanoparticle solutions recorded immediately after synthesis. A sharp peak at 420 nm (Figure 1 b, Figure S1) confirms the presence of sub‐nanometre clusters, containing approximately 11 Au atoms, indicating the quantized electronic structure, while other spectra contain a broad peak at higher wavelengths (500–600 nm), due to polydispersity of the Au nanoparticle size distribution.[^12a^, ^16^] The gold clusters formed are stable for at least 30 days. To validate these results, the size of the Au clusters is confirmed by high resolution transmission electron microscopy (HRTEM). The HRTEM micrograph and corresponding particle size distribution (Figure 1 c, d) demonstrates that the average core diameter of the phosphine stabilised gold clusters is ca. 0.8 nm. This methodology does not require any complicated purification of the Au^I^ precursor and directly produces in situ phosphine‐stabilised Au clusters.

**Figure 2:**
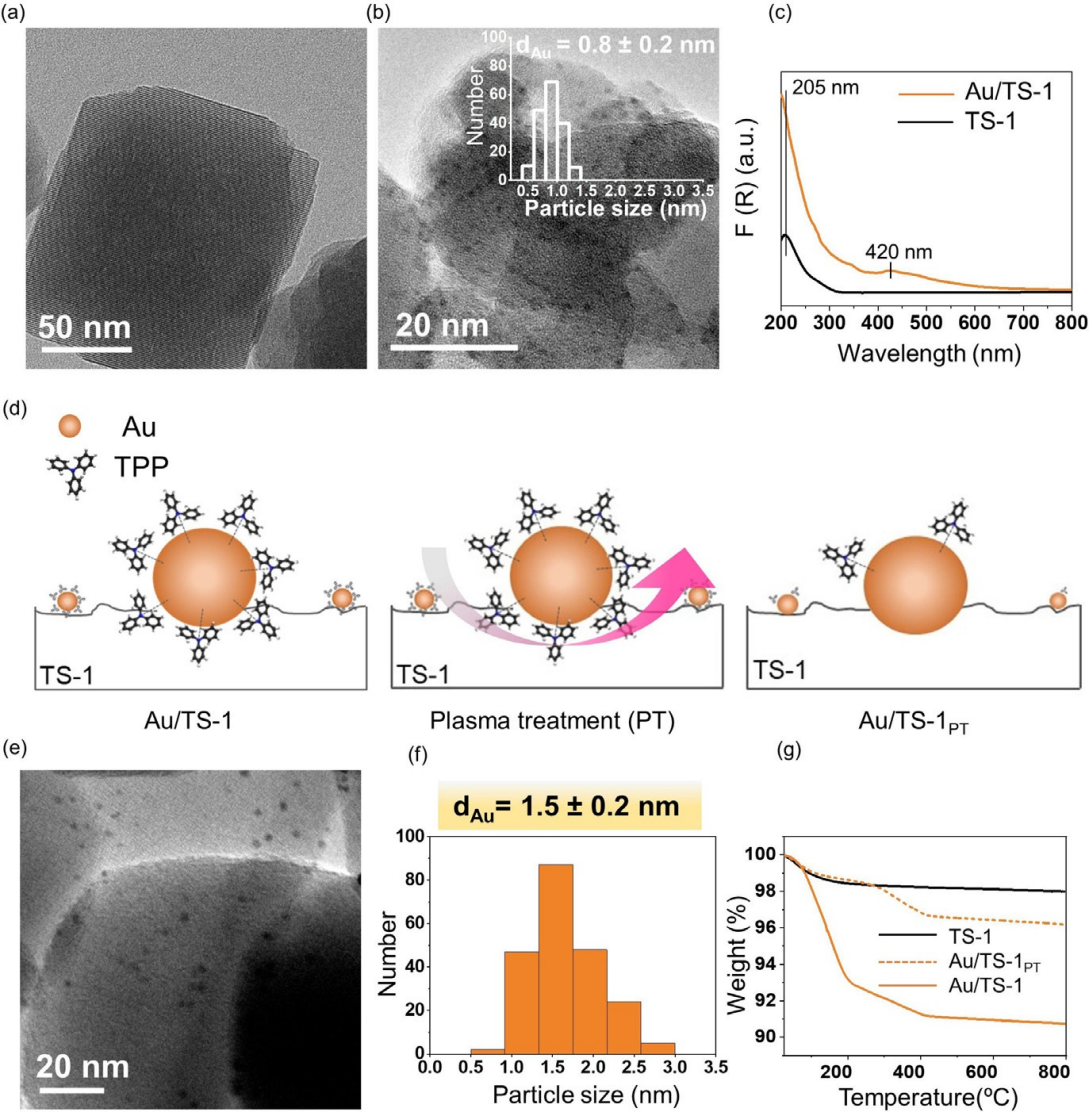
Characterisation of immobilised gold clusters onto TS‐1 (Au/TS‐1) and non‐thermal plasma removal. a) TEM image of TS‐1. b) TEM image of Au/TS‐1 (inset shows the particle size distribution histogram). c) DR‐UV/Vis of TS‐1 and Au/TS‐1. d) Schematic illustration of non‐thermal oxygen plasma treatment on Au/TS‐1. e) TEM of Au/TS‐1_PT_. f) Corresponding Au particle size distribution histogram. g) Thermogravimetric analysis (TGA) of TS‐1, Au/TS‐1 and Au/TS‐1_PT_.

The titanium silicalite‐1 (TS‐1, Si/Ti=60) zeolite support is hydrothermally synthesised according to Nijhuis et al.^[1a]^ Figure 2 a shows a typical HRTEM image of TS‐1, indicating a highly crystalline material. The nitrogen sorption isotherm (Figure S2) is of IUPAC Type I, characteristic for purely microporous materials, and both micropore volume and BET surface area are typical for TS‐1 zeolites.^[17]^ The UV/Vis spectrum shown in Figure 2 c reveals that the titanium present in the zeolite sample is mostly in tetrahedral coordination, as evident in the peaks at 205 nm attributed to tetrahedral titanium species.^[18]^

Sub‐nanometre Au clusters are prepared in ethanol and mixed with TS‐1 in a round bottom flask to obtain 1 wt. % Au loading. The ethanol solvent is evaporated, and an orange coloured powder is obtained after drying and purification. The final size of the clusters after immobilisation is calculated using HRTEM and the average particle size of the clusters remains ca. 0.8 nm after immobilisation, as illustrated in Figure 2 b. After deposition of the gold clusters on the TS‐1, no significant structural changes in the zeolite are evident in XRD (Figure S3) and DR‐UV/Vis spectroscopy (Figure 2 c). The DR‐UV/Vis spectrum shows a sharp peak at 420 nm, indicating the presence of sub‐nanometre gold clusters, and the lack of a surface plasmon resonance band at 500–600 nm further confirms the absence of any larger gold particles (aggregates).[^12a^, ^16^] Furthermore, the colour of the powder does not change to purple or black after immobilisation, as shown in Figure S4.^[19]^

The Au clusters are stabilised by triphenylphosphine ligands and, in order to increase accessibility to the Au surface, it is important to remove the stabilising agent. These capping agents prevent the aggregation of the nanoparticles but at the same time block the access to the active sites.^[20]^ The traditional routes employed for the removal of ligands are thermal and oxidative treatments. These techniques induce inevitable mobility of metal atoms on the support, which leads to an increase in particle size and loss of monodispersity.^[21]^ This makes it very difficult to control catalytic activity through a specific particle size. It is well known that small Au nanoparticles, and particularly those with a core diameter below 5 nm, are crucial to achieve catalytic activity in propylene epoxidation.[^7a^, ^22^] Thus, in an effort to avoid harsh ligand removal methods, we demonstrate a simple way to remove ligands by using a non‐thermal O_2_ plasma technique that does not induce an increase in the size of the Au nanoparticles.^[23]^ Figure 2 d schematically illustrates the O_2_ plasma removal procedure for Au/TS‐1 materials. Au/TS‐1 powder is placed in a vacuum chamber and irradiated with a plasma for 30 minutes. The energetic O_2_ plasma species remove the triphenylphosphine ligands under vacuum. To monitor any particle size changes of Au/TS‐1 after plasma treatment, particle size is measured via TEM. A TEM image of Au/TS‐1_PT_ is shown in Figure 2 e and the corresponding particle size distribution (Figure 2 f) confirms that the average particle size has increased slightly to 1.5 nm, which is considered an appropriate size for catalytic applications.^[24]^

It is evident from the TEM images that the particles remain uniformly dispersed onto the zeolite after the ligand removal. To quantify the amount of TPP removed from the sample, thermogravimetric analysis (TGA) is performed (Figure 2 g). The TGA curves show that the total weight loss of the plasma treated Au/TS‐1 (1.8 %) is lower than that of the untreated sample (7.3 %), further suggesting that 74 wt. % of the total amount of ligands have been removed. The tetrahedrally and octahedrally coordinated Ti sites in the TS‐1 framework remain unchanged after ligand removal, which is confirmed using DR‐UV/Vis spectroscopy (Figure S5). The UV/Vis spectrum also shows the peak at 420 nm, which indicates that the characteristic absorption band of small gold NPs remains unchanged after the plasma treatment. For comparison with thermal methods, a Au/TS‐1 catalyst was also calcined at 300 °C in air to remove triphenylphosphine ligands. Figure S6a illustrates the TGA curve, showing 80 % ligand removal after calcination. DR‐UV/Vis spectroscopy (Figure S6b) and TEM (Figure S6c,d) further confirm that the particle size increases to 11.7±3.7 nm, which shows that high temperature leads to an increased particle size, due to agglomeration of the gold nanoparticles. Hence, it can be concluded from the employed characterisation techniques that the non‐thermal plasma treatment removes a substantial amount of bound TPP ligands from the gold nanoparticles without greatly altering their particle size and morphology, both factors having important implications on catalytic performance.^[25]^

**Figure 3:**
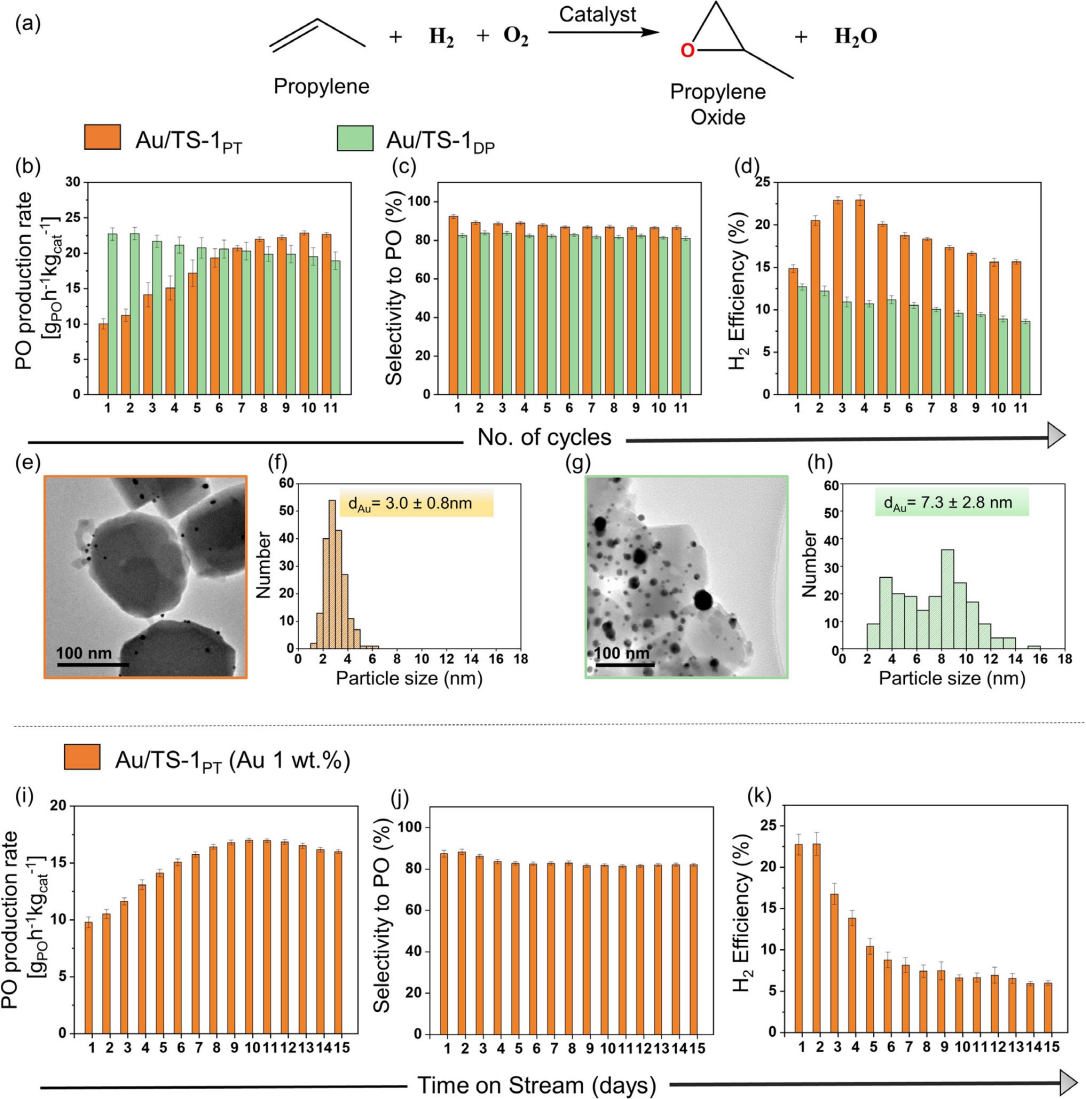
Catalytic performance of Au/TS‐1 catalysts. a) Direct propylene epoxidation reaction. Comparison studies of Au/TS‐1_PT_ and Au/TS‐1_DP_ catalyst. b) Propylene oxide (PO) production rate. c) PO selectivity. d) Hydrogen efficiency. e) TEM image of spent Au/TS‐1_PT_ catalyst. f) Particle size distribution histogram of spent Au/TS‐1_PT_ catalyst. g) TEM image of spent Au/TS‐1_DP_ catalyst. h) Particle size distribution histogram of spent Au/TS‐1_DP_ catalyst. Stability test for Au/TS‐1_PT_ catalyst. i) Propylene oxide (PO) production rate. j) PO selectivity. k) Hydrogen efficiency.

The Au/TS‐1_PT_ (plasma treated, 1 wt. % Au) catalyst is tested (Figure 3 a) in a quartz tubular reactor for 5‐hour cycles at a reaction temperature of 200 °C. The catalyst is regenerated after every cycle by heating at 300 °C under 10 % oxygen in helium for 1 h to remove organic species which accumulate on its surface over time.[^8b^, ^26^] For comparison, a more conventional Au/TS‐1 (1 wt. % Au) catalyst is prepared as well, in which Au is directly dispersed on the support using the deposition precipitation (DP) method (characterisation data in Figure S7) and tested under the same reaction conditions.^[1a]^ The catalytic performance of both catalysts is compared in Figure 3 b–d. In Figure 3 b, the Au/TS‐1_DP_ catalyst shows a higher initial PO formation rate (22 g_PO_ h^−1^ kg_cat_^−1^) compared to the Au/TS‐1_PT_ (10 g_PO_ h^−1^ kg_cat_^−1^), which could be because of the ligand‐free Au nanoparticles incorporated into active Ti^IV^ sites. The initially low PO formation rate of Au/TS‐1_PT_ can be attributed to the lower accessibility of the active sites, due to partial coverage of the Au surface with TPP ligands; however, as the time‐on‐stream (TOS) increases, there is an appreciable increase of the PO formation rate (from 10 to 20 g_PO_ h^−1^ kg_cat_^−1^), which reaches a maximum of 22.5 g_PO_ h^−1^ kg_cat_^−1^ (propylene single pass conversion ca. 1.3 %, hydrogen single pass conversion ca. 8.2 %, oxygen single pass conversion ca. 6.2 %) by the 11th cycle. As the reaction temperature is 200 °C, the residual protecting ligands present in the catalyst's vicinity are removed during reaction, since the decomposition temperature of triphenylphosphine is around 180 °C.^[16]^ The TPP removal under reaction conditions leads to increased accessibility of Au‐Ti active sites, which results in higher activity. On the other hand, the Au/TS‐1_DP_ catalyst starts to deactivate with increasing time‐on‐stream, with the rate decreasing from 22 to 18.8 g_PO_ h^−1^ kg_cat_^−1^ (propylene single pass conversion ca. 1.2 % hydrogen single pass conversion ca. 11.6 %, oxygen single pass conversion ca. 7.7 %).^[27]^

Apart from the activity, the PO selectivity and H_2_ efficiency are the two other important factors defining the catalytic performance.^[28]^ The selectivity towards PO for both catalysts are shown in Figure 3 c. The Au/TS‐1_DP_ catalyst exhibits PO selectivity of ca. 82 %, whereas the Au/TS‐1_PT_ catalyst shows a higher selectivity of ca. 88 % over the time‐on‐stream. Figure 3 d shows the hydrogen efficiency for both catalysts, and it can be easily seen that Au/TS‐1_PT_ has, overall, a much higher H_2_ efficiency than the traditional catalyst. The initial increase of H_2_ efficiency from 15 to 23 % in the case of Au/TS‐1_PT_ is related to the availability of Au active sites over time, which are partially obstructed in the beginning. On the contrary, the H_2_ efficiency value constantly drops for Au/TS‐1_DP_ from 12 to 8 %, indicating that a large amount of H_2_ is directly converted to water, hence making the process economically unviable. It can be concluded that Au/TS‐1_PT_ exhibits better hydrogen efficiency, high PO selectivity, and remarkably improved stability between subsequent cycles, as compared to the Au/TS‐1_DP_ catalysts.

Two factors contribute to deactivation in this reaction: one is the adsorption of carbonates/carboxylate species on the catalyst surface, which is easily reversible by periodic regenerations to remove such species, while the second is the sintering of gold nanoparticles to form larger aggregates, which is an irreversible phenomenon.[^5a^, ^29^] Although most of the deactivating species are removed by regeneration, sintering can still occur during the reaction, which is irreversible and thus could account for a decreasing activity. To assess this, both spent catalysts are analysed using TEM (Figure 3 e–h) to observe changes in catalyst morphology. It is evident from Figure 3 g, h that gold particles in the Au/TS‐1_DP_ catalyst are sintered to a higher degree over the total time‐on‐stream, with an average particle size of ca. 7.3 nm. On the other hand, the average particle size of the gold in the spent Au/TS‐1_PT_ catalyst (Figure 3 e, f) is only ca. 3 nm and large agglomerates are not observed, which is one of the possible reasons for the enhanced catalytic stability. Therefore, the main reason behind the decreased activity can be attributed to the sintering of gold particles, due to the absence of protecting agents, which play an important role in stabilisation during the catalyst synthesis.^[30]^ The results show that Au/TS‐1_PT_ outperforms the Au/TS‐1_DP_ in terms of activity and hydrogen efficiency over a longer time in the direct gas‐phase epoxidation of propylene.

In the above study, it is observed that the Au/TS‐1_PT_ catalyst possesses improved catalytic performance compared to the Au/TS‐1_DP_ catalyst. The conventional catalyst gradually deactivates over time‐on‐stream, losing activity, selectivity, and hydrogen efficiency, in accordance with previous reports in the literature.[^6e^, ^29a^] In contrast, the Au/TS‐1_PT_ does not show any deactivation under the same reaction conditions. Subsequently, Au/TS‐1_PT_ is tested for a longer time‐on‐stream, without the periodical regeneration, to evaluate its durability and stability, which are crucial indicators for industrial catalysts. The catalytic results of Au/TS‐1_PT_ (1 wt. % Au) are illustrated in Figure 3 i–k. The propylene epoxidation involves in situ peroxo species formation from H_2_ and O_2_, which can either react with propylene to form PO or can decompose or be hydrogenated to produce water. Figure 3 i reveals that there is an initial increase in PO production rate, related to the higher accessibility of Au sites, due to the slow removal of the bound ligands at 200 °C that facilitates the formation of more peroxo species. In small gold nanoparticles most of the atoms are on the periphery, lying in a close proximity to Ti^4+^ sites, allowing easier access to reaction intermediates to produce PO.^[31]^ PO production rate reaches a maximum on day 11 (16.9 g_PO_ h^−1^ kg_cat_^−1^, propylene single pass conversion ca. 1.1 % hydrogen single pass conversion ca. 12.7 %, oxygen single pass conversion ca. 9.4 %) and then a slight decrease in the PO production rate is evident. The catalyst demonstrates a higher PO selectivity ca. 84 % with a smaller amount of side products (Figure 3 j) than the untreated Au/TS‐1_DP_ catalyst.^[1a]^ The H_2_ efficiency (Figure 3 k) decreases from 23 to 10 % in 6 days. As the time‐on‐stream increases, a higher number of Au sites become available, which produces more peroxo species, causing an increase in both PO formation and water formation. After some time, H_2_O_2_ formed on the larger Au nanoparticles travels a longer way to nearby Ti^4+^ sites, where it rapidly decomposes to form more H_2_O, resulting in lower hydrogen efficiency values.[^22b^, ^31^] The epoxidation rate and the hydrogen efficiency are determined by a delicate balance between the rate of peroxo formation and its consumption by the epoxidation reaction. If the peroxo formation becomes too fast, the epoxidation cannot keep up and the hydrogen efficiency drops. Lowering the gold loading is, therefore, a highly effective way to further increase the hydrogen efficiency, although at the expense of activity.^[32]^ The optimum gold loading and its spatial location should be determined based on an economic evaluation of the process.^[33]^ Furthermore, the productivity of the catalyst could be enhanced by using core–shell structures, alkali metal promoters or employing bimetallic nanoparticles.[^8d^, ^34^] From the previous discussion, it is apparent that there is no significant decrease in activity and PO selectivity during a long lifetime test without regeneration, which indicates the remarkable stability of these catalysts. Notably, there is no change in the TS‐1 structure during the reaction, which is evident from the TEM and DR‐UV/Vis (Figure S8, S9) measured after the reaction.

It is well understood from previous reports that Au catalysts suffer from deactivation because of the agglomeration of particles and accumulation of carbonaceous species on the catalyst surface over the course of a reaction.[^29a^, ^35^] However, the reported Au/TS‐1_PT_ material shows remarkable stability and high selectivity towards PO formation over the time on stream. To understand the mechanism(s) behind the enhanced stability of Au/TS‐1_PT_, we utilize multiple characterisation techniques, such as X‐ray photoelectron spectroscopy (XPS), ^31^P MAS NMR spectroscopy, TGA, DR‐UV/Vis, and TEM to investigate changes in the catalyst structure and the deactivation mechanism(s) during the propylene epoxidation reaction.

**Figure 4:**
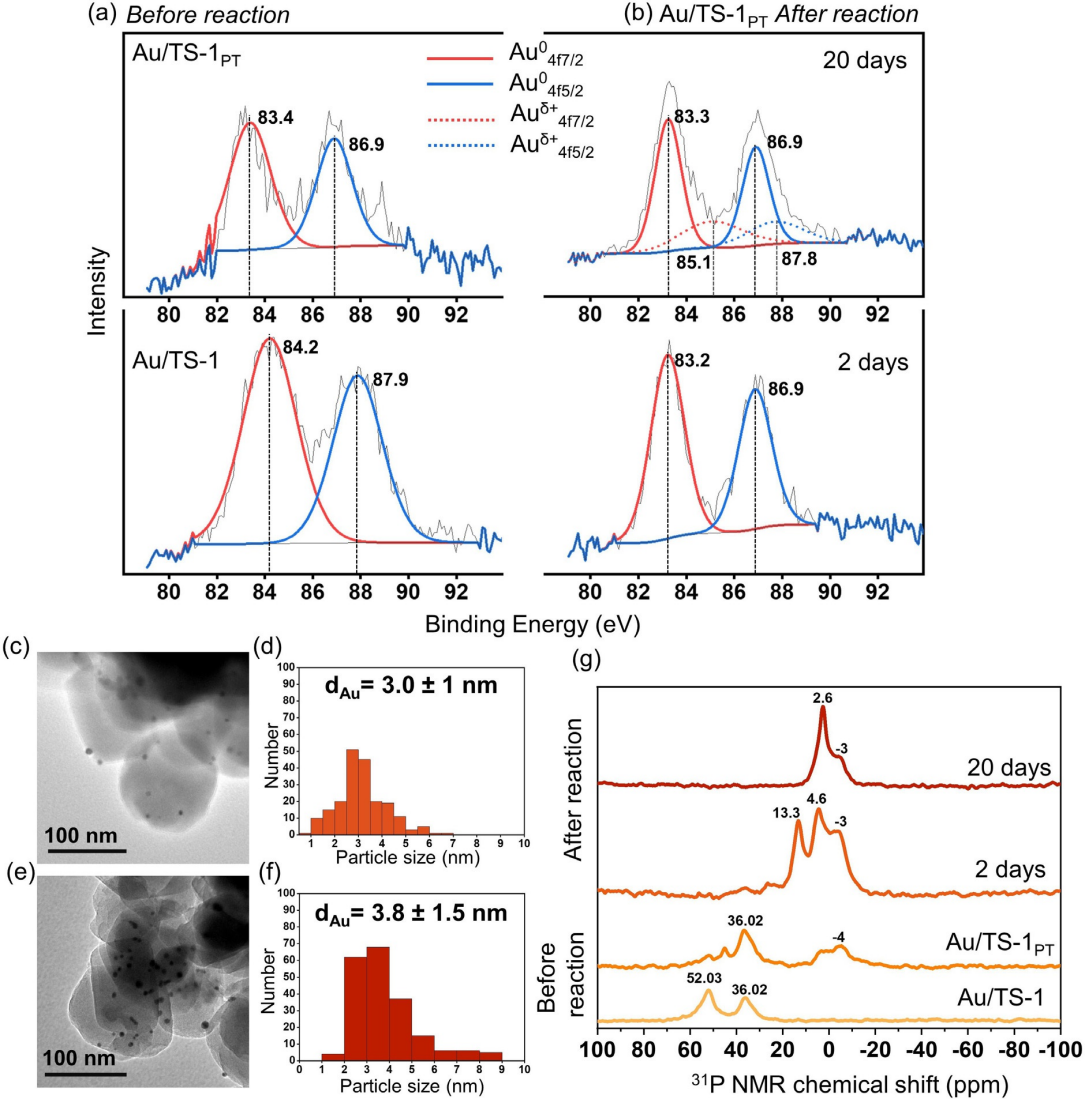
Investigations to elucidate structure‐stability relationships. a) XPS spectra of gold for Au/TS‐1 before reaction. b) XPS spectra of gold for Au/TS‐1_PT_ after reaction. c) TEM of Au/TS‐1_PT_ after 2 days of reaction. d) Particle size distribution histogram of (c). e) TEM of Au/TS‐1_PT_ after 20 days of reaction. f) Particle size distribution histogram of (e). g) ^31^P MAS NMR of Au/TS‐1_PT_ before and after reaction.

X‐ray photoelectron spectroscopy (XPS) investigates changes in the chemical state and the size of gold clusters deposited on TS‐1, since the 4f_7/2_ orbital of gold provides a sensitive measure of its electronic state and the full‐width‐half‐maximum (FWHM) of the gold peak is related to its particle size.^[36]^ The 4f_7/2_ signal of the untreated Au/TS‐1 sample is ca. 84.2 eV (Figure 4 a), a value characteristic of gold in metallic state (Au^0^).[^36^, ^37^] After treatment with oxygen plasma (Figure 4 a), the 4f_7/2_ peak of gold shifts to ca. 83.4 eV, indicating the coexistence of metallic gold and undecomposed gold‐phosphine complex (Au(PPh_3_)(Cl)).^[38]^ In the case of Au/TS‐1_PT_ that had twenty days on‐stream, additional peaks of gold appear at ca. 85.1 and ca. 87.8 eV, revealing the presence of oxidised gold species (Au^δ+^, 0<δ<3).^[39]^ The atomic percentage of oxidised gold is ca. 41 %, suggesting the partial oxidation of gold clusters after twenty days of continuous reaction. Furthermore, a decrease in the value of the FWHM of the 4f_7/2_ peak of gold is observed (Table 1) for Au/TS‐1_PT_ after reaction, indicating the formation of agglomerates.^[36]^ This interpretation is verified by DR‐UV/Vis spectroscopy (Figure S9), where a surface plasmon band at 520 nm appears for both samples, again indicating a larger particle size. TEM images (Figure 4 c, d) show that the size of the Au particle is ca. 3 nm after 2 days and reaches ca. 4 nm (Figure 4 e, f) after 20 days of reaction. This partial agglomeration and oxidation of gold clusters results in a slight decrease in catalytic activity^[40]^ as the time‐on‐stream increases (Figure 3 i–k).^[41]^ Furthermore, the comparison of Ti 2p spectra of untreated and post reaction Au/TS‐1 samples (Figure S10) reveals no shift in the binding energy, indicating the high stability of Ti in such oxidizing environment.^[42]^ Titania plays a crucial role in the overall stability of Au/TS‐1 catalyst, since there is strong interaction between Au and Ti via adsorption of Au nanoparticles on Ti defect sites of the TS‐1 lattice.[^42^, ^43^] These Ti sites act as nucleating sites for Au nanoparticles (in the range of 1–10 nm) resulting in a highly stable Au/TS‐1 catalyst.^[44]^

**Table 1:** Position and FWHM of Au 4f_7/2_ XPS peaks of all Au/TS‐1 samples tested.^[a]^

	Au/TS‐1		Au/TS‐1_PT_		Au/TS‐1_PT_ 2 days reaction		Au/TS‐1_PT_ 20 days reaction	
	B.E.	FWHM	B.E.	FWHM	B.E.	FWHM	B.E.	FWHM
Au4f_7/2_	84.2	2.64	83.4	2.03	83.2	1.66	83.3	1.41

[a] All values are in eV.

Additionally, the treatment of a Au/TS‐1 sample with oxygen plasma alters its surface properties. The untreated Au/TS‐1 sample has a phosphorus peak at ca. 131.8 eV (Figure S11a), assigned to phosphine ligands bonded to the gold cluster.^[22a]^ However, this phosphorus peak shifts to ca. 133 eV (Figure S11b–d) after treatment of the sample with oxygen plasma, demonstrating the formation of phosphorus oxide bonded on the surface of TS‐1, due to the dislodging of phosphine ligands from the gold clusters and their subsequent binding and oxidation via interaction with the surface of the metal oxide support.^[22a]^ ^31^P MAS NMR spectroscopy is applied to analyse the nature of the phosphorus on TS‐1 in the catalyst, before and after the reaction, to better comprehend the structure‐stability relationship (Figure 4 g). The ^31^P NMR spectra of all the Au/TS‐1 samples are shown in Figure 4 g. As depicted, the Au/TS‐1 sample exhibits strong signals at 52 and 36 ppm, which are ascribed to surface bound PPh_3_ to Au and a phosphine‐gold complex, Au(PPh_3_)Cl, respectively.^[45]^ After plasma treatment, there is a decrease in the intensity of the signal observed at 52 ppm with simultaneous appearance of a peak at −4 ppm, corresponding to physiosorbed phosphine,^[46]^ which indicates that some fraction of the bound PPh_3_ is moved onto the TS‐1 support. The presence of a small amount of ligand in the catalyst is a plausible reason for the long induction period for the Au/TS‐1_PT_ catalyst. After 2 days of reaction, the peak at 36 ppm (Au(PPh_3_)Cl) completely vanishes, which is attributed to the decomposition of the ligand within the reactor at high reaction temperature. Additionally, the signals appearing at ca. 13.3 and −3 ppm are linked to the phosphine species migrated to the support.[^46^, ^47^] The peak at 4.6 ppm is ascribed to some phosphate species.^[48]^ However, after 20 days of reaction, most of the phosphine ligands are oxidised to phosphates, corresponding to the peak at 2.6 ppm, also confirmed by XPS analysis (Figure S11c, d). This observation is corroborated by TGA data (Figure S12), where the total weight loss for the catalyst after 2 days of reaction is double that of the spent catalyst after 20 days. This indicates that most of the phosphine species have decomposed, while the remaining phosphine species migrated and adsorbed onto the TS‐1, and slowly oxidise to phosphates with increasing time‐on‐stream. It has been previously reported that phosphate grafting onto TS‐1 can be used to enhance epoxidation.^[49]^ Therefore, the increasing trend in the PO production rate is linked to more accessible Au‐Ti sites with the gradual release of the bound phosphine species at 200 °C from the Au surface. The subsequent decrease in the PO production rate after 11 days can be associated to partial aggregation of Au particles caused by the loss of ligands. Overall, the catalyst exhibited a significantly prolonged lifetime compared to the conventional Au/TS‐1 catalyst, which is hypothesised to be due to the stabilising effect of residual phosphine ligands on the gold particles. The residual bound phosphine ligands prevent sintering and significantly improve its stability in the harsh reaction environment. Moreover, the importance of gold particle size and phosphine ligand is further confirmed on testing a calcined Au/TS‐1 catalyst, where 80 % of the ligand was removed prior to the reaction. The calcined Au/TS‐1 samples showed a lower PO production rate of 10.4 g_PO_ h^−1^ kg_cat_^−1^ (propylene conversion 0.9 % hydrogen single pass conversion ca. 8.2 %, oxygen single pass conversion ca. 5 %) after 30 h of reaction (Figure S13a). The calcined catalyst exhibits a low PO selectivity of 56.9 % (Figure S13b) with propanal as the other primary product, along with CO_2_ and water. This lower PO selectivity is linked to the much larger gold nanoparticles that favour direct combustion. The calcined catalyst shows very low H_2_ efficiency values of ca. 3 % (Figure S13c) initially, which is indicative of more water formation accompanied by low PO production. As the reaction proceeds, this value increases to ca. 6 % on 20 h time‐on‐stream.

## Conclusion

Sub‐nanometre gold clusters with a core diameter of approximately 0.8 nm, stabilised by phosphine ligands, are synthesised using a facile one‐pot methodology. The clusters can be easily immobilised onto the support with minimal change in particle size. A fast technique using non‐thermal O_2_ plasma has been developed to remove around 74 % of phosphine ligands, leading to a highly stable catalyst. The catalyst prepared using this approach demonstrates better catalytic performance in direct gas phase propylene epoxidation based on PO selectivity of ca. 89 % and stability over 20 days. Comparing these results to traditional Au/TS‐1 catalysts in direct gas‐phase epoxidation,^[29a]^ there is significant improvement in overall lifetime, increased selectivity, higher hydrogen utilisation, and less nanoparticle sintering. Triphenylphosphine plays an important role as a sacrificial labile ligand in preventing nanoparticle agglomeration, while keeping the overall particle size small (<5 nm) and simultaneously improving the size specific catalytic activity. Apart from these phosphine ligands, an additional factor contributing to the enhanced stability of Au nanoparticles during the epoxidation reaction is the presence of Ti defect sites, which act as nucleating sites for Au nanoparticles, preventing their oxidation and improving the stability of Au/TS‐1.[^42^, ^43^, ^44^] Although this work focused on propylene epoxidation, the insights provided herein can be used to rationally design stable catalysts with a longer lifetime for other reactions, which is an important aspect in the design of catalysts for industrial applications.

## Conflict of interest

The authors declare no conflict of interest.

## Supporting information

As a service to our authors and readers, this journal provides supporting information supplied by the authors. Such materials are peer reviewed and may be re‐organized for online delivery, but are not copy‐edited or typeset. Technical support issues arising from supporting information (other than missing files) should be addressed to the authors.

Supporting Information

## References

[1a] T. A. Nijhuis, B. J. Huizinga, M. Makkee, J. A. Moulijn, Ind. Eng. Chem. Res. 1999, 38, 884–891;

[1b] T. A. Nijhuis, M. Makkee, J. A. Moulijn, B. M. Weckhuysen, Ind. Eng. Chem. Res. 2006, 45, 3447–3459;

[1c] Y. Lei, F. Mehmood, S. Lee, J. Greeley, B. Lee, S. Seifert, R. E. Winans, J. W. Elam, R. J. Meyer, P. C. Redfern, Science 2010, 328, 224–228; 20378815 10.1126/science.1185200

[1d] J. Teržan, M. Huš, B. Likozar, P. Djinović, ACS Catal. 2020, 10, 13415–13436;

[1e] M. Ojeda, E. Iglesia, Chem. Commun. 2009, 352–354. 10.1039/b813589d 19209326

[2] T. Dina, A. S. Bescond, Chemical Economics Handbook: Propylene Oxide, IHS Markit, 2019.

[3a] S. J. Khatib, S. T. Oyama, Catal. Rev. 2015, 57, 306–344;

[3b] J. Huang, M. Haruta, Res. Chem. Intermed. 2012, 38, 1–24;

[3c] Z. Li, L. Gao, X. Zhu, W. Ma, X. Feng, Q. Zhong, ChemCatChem 2019, 11, 5116–5123.

[4a] V. Russo, R. Tesser, E. Santacesaria, M. Di Serio, Ind. Eng. Chem. Res. 2013, 52, 1168–1178;

[4b] J. K. Edwards, E. Ntainjua, A. F. Carley, A. A. Herzing, C. J. Kiely, G. J. Hutchings, Angew. Chem. Int. Ed. 2009, 48, 8512–8515; 10.1002/anie.200904115 19802864

[4c] G. J. Hutchings, R. Lewis, ChemCatChem 2019, 11, 298–308.

[5a] T. A. Nijhuis, J. Chen, S. M. Kriescher, J. C. Schouten, Ind. Eng. Chem. Res. 2010, 49, 10479–10485;

[5b] T. Ishida, T. Murayama, A. Taketoshi, M. Haruta, Chem. Rev. 2020, 120, 464–525; 31820953 10.1021/acs.chemrev.9b00551

[5c] M. Lin, C. Xia, B. Zhu, H. Li, X. Shu, Chem. Eng. J. 2016, 295, 370–375.

[6a] T. Hayashi, K. Tanaka, M. Haruta, J. Catal. 1998, 178, 566–575;

[6b] B. S. Uphade, T. Akita, T. Nakamura, M. Haruta, J. Catal. 2002, 209, 331–340;

[6c] M. Haruta, B. Uphade, S. Tsubota, A. Miyamoto, Res. Chem. Intermed. 1998, 24, 329–336;

[6d] A. Sinha, S. Seelan, S. Tsubota, M. Haruta, Top. Catal. 2004, 29, 95–102;

[6e] X. Feng, N. Sheng, Y. Liu, X. Chen, D. Chen, C. Yang, X. Zhou, ACS Catal. 2017, 7, 2668–2675;

[6f] M. Haruta, Nature 2005, 437, 1098–1099; 16237427 10.1038/4371098a

[6g] J. Huang, T. Akita, J. Faye, T. Fujitani, T. Takei, M. Haruta, Angew. Chem. Int. Ed. 2009, 48, 7862–7866; 10.1002/anie.200903011 19757468

[6h] G. J. Hutchings, J. Catal. 1985, 96, 292–295;

[6i] G. J. Hutchings, Catal. Today 2005, 100, 55–61;

[6j] G. J. Hutchings, M. Brust, H. Schmidbaur, Chem. Soc. Rev. 2008, 37, 1759–1765; 18762825 10.1039/b810747p

[6k] T. Takei, T. Akita, I. Nakamura, T. Fujitani, M. Okumura, K. Okazaki, J. Huang, T. Ishida, M. Haruta, Adv. Catal. 2012, 55, 1–126;

[6l] C. Qi, Gold Bull. 2008, 41, 224–234.

[7a] Z. Lu, M. Piernavieja-Hermida, C. H. Turner, Z. Wu, Y. Lei, J. Phys. Chem. C 2018, 122, 1688–1698;

[7b] T. A. Nijhuis, T. Visser, B. M. Weckhuysen, Angew. Chem. Int. Ed. 2005, 44, 1115–1118; 10.1002/anie.200462043 15643664

[7c] G. Wang, Y. Cao, Z. Zhang, J. Xu, M. Lu, G. Qian, X. Duan, W. Yuan, X. Zhou, Ind. Eng. Chem. Res. 2019, 58, 17300–17307.

[8a] J. Huang, T. Takei, H. Ohashi, M. Haruta, Appl. Catal. A 2012, 435–436, 115–122;

[8b] E. Sacaliuc, A. Beale, B. Weckhuysen, T. Nijhuis, J. Catal. 2007, 248, 235–248;

[8c] C. Qi, J. Huang, S. Bao, H. Su, T. Akita, M. Haruta, J. Catal. 2011, 281, 12–20;

[8d] Y. Ren, X. Sun, J. Huang, L. Zhang, B. Zhang, M. Haruta, A.-H. Lu, Ind. Eng. Chem. Res. 2020, 59,8155–8163;

[8e] Y. Hong, L. Ke, Z. Li, J. Huang, G. Zhan, Y. Zhou, D. Sun, J. Zhang, Q. Li, Catal. Lett. 2020, 150, 1798–1811.

[9] H. O. Otor, J. B. Steiner, C. García-Sancho, A. C. Alba-Rubio, ACS Catal. 2020, 10, 7630–7656.

[10] C. H. Bartholomew, Appl. Catal. A 2001, 212, 17–60.

[11a] G. Mul, A. Zwijnenburg, B. van der Linden, M. Makkee, J. A. Moulijn, J. Catal. 2001, 201, 128–137;

[11b] Z. Li, W. Ma, Q. Zhong, Catal. Lett. 2020, 150,1856–1864.

[12a] N. De Silva, J.-M. Ha, A. Solovyov, M. M. Nigra, I. Ogino, S. W. Yeh, K. A. Durkin, A. Katz, Nat. Chem. 2010, 2, 1062–1068; 21107371 10.1038/nchem.860

[12b] P. Trogadas, M. M. Nigra, M.-O. Coppens, New J. Chem. 2016, 40, 4016–4026.

[13] R. Zanella, L. Delannoy, C. Louis, Appl. Catal. A 2005, 291, 62–72.

[14a] N. Yap, J. Catal. 2004, 226, 156–170;

[14b] M. Sankar, Q. He, R. V. Engel, M. A. Sainna, A. J. Logsdail, A. Roldan, D. J. Willock, N. Agarwal, C. J. Kiely, G. J. Hutchings, Chem. Rev. 2020, 120, 3890–3938. 32223178 10.1021/acs.chemrev.9b00662 PMC7181275

[15] W. W. Weare, S. M. Reed, M. G. Warner, J. E. Hutchison, J. Am. Chem. Soc. 2000, 122, 12890–12891.

[16] Y. Liu, H. Tsunoyama, T. Akita, T. Tsukuda, J. Phys. Chem. C 2009, 113, 13457–13461.

[17] M. Thommes, K. Kaneko, A. V. Neimark, J. P. Olivier, F. Rodriguez-Reinoso, J. Rouquerol, K. S. Sing, Pure Appl. Chem. 2015, 87, 1051–1069.

[18] P. Wu, T. Tatsumi, T. Komatsu, T. Yashima, J. Phys. Chem. B 2001, 105, 2897–2905.

[19] M. Brust, C. J. Kiely, D. Bethell, D. J. Schiffrin, J. Am. Chem. Soc. 1998, 120, 12367–12368.

[20a] S. M. Ansar, C. L. Kitchens, ACS Catal. 2016, 6, 5553–5560;

[20b] Z. Niu, Y. Li, Chem. Mater. 2014, 26, 72–83.

[21a] S. Tsubota, T. Nakamura, K. Tanaka, M. Haruta, Catal. Lett. 1998, 56, 131–135;

[21b] J. A. Lopez-Sanchez, N. Dimitratos, C. Hammond, G. L. Brett, L. Kesavan, S. White, P. Miedziak, R. Tiruvalam, R. L. Jenkins, A. F. Carley, D. Knight, C. J. Kiely, G. J. Hutchings, Nat. Chem. 2011, 3, 551. 21697877 10.1038/nchem.1066

[22a] D. P. Anderson, J. F. Alvino, A. Gentleman, H. A. Qahtani, L. Thomsen, M. I. J. Polson, G. F. Metha, V. B. Golovko, G. G. Andersson, Phys. Chem. Chem. Phys. 2013, 15, 3917–3929; 23400365 10.1039/c3cp44005b

[22b] X. Feng, X. Duan, G. Qian, X. Zhou, D. Chen, W. Yuan, J. Catal. 2014, 317, 99–104;

[22c] Z. Li, J. Zhang, D. Wang, W. Ma, Q. Zhong, J. Phys. Chem. C 2017, 121, 25215–25222.

[23a] J. H. Maurer, L. González-García, B. Reiser, I. Kanelidis, T. Kraus, ACS Appl. Mater. Interfaces 2015, 7, 7838–7842; 25838194 10.1021/acsami.5b02088

[23b] X. Liu, C.-Y. Mou, S. Lee, Y. Li, J. Secrest, B. W.-L. Jang, J. Catal. 2012, 285, 152–159;

[23c] E. W. Elliott III , R. D. Glover, J. E. Hutchison, ACS Nano 2015, 9, 3050–3059. 25727562 10.1021/nn5072528

[24a] S. Lee, L. M. Molina, M. J. Lopez, J. A. Alonso, B. Hammer, B. Lee, S. Seifert, R. E. Winans, J. W. Elam, M. J. Pellin, S. Vajda, Angew. Chem. Int. Ed. 2009, 48, 1467–1471; 10.1002/anie.200804154 19152388

[24b] J. Huang, E. Lima, T. Akita, A. Guzmán, C. Qi, T. Takei, M. Haruta, J. Catal. 2011, 278, 8–15.

[25a] N. Lopez, T. Janssens, B. Clausen, Y. Xu, M. Mavrikakis, T. Bligaard, J. K. Nørskov, J. Catal. 2004, 223, 232–235;

[25b] A. S. K. Hashmi, G. J. Hutchings, Angew. Chem. Int. Ed. 2006, 45, 7896–7936; 10.1002/anie.200602454 17131371

[25c] A. Corma, P. Concepción, M. Boronat, M. J. Sabater, J. Navas, M. J. Yacaman, E. Larios, A. Posadas, M. A. López-Quintela, D. Buceta, Nat. Chem. 2013, 5, 775–781. 23965680 10.1038/nchem.1721

[26] T. Nijhuis, B. Weckhuysen, Catal. Today 2006, 117, 84–89.

[27] W.-S. Lee, L.-C. Lai, M. Cem Akatay, E. A. Stach, F. H. Ribeiro, W. N. Delgass, J. Catal. 2012, 296, 31–42.

[28] J. Huang, M. Haruta, Res. Chem. Intermed. 2012, 38, 1–24.

[29a] W.-S. Lee, M. Cem Akatay, E. A. Stach, F. H. Ribeiro, W. N. Delgass, J. Catal. 2012, 287, 178–189;

[29b] T. A. Nijhuis, E. Sacaliuc, B. M. Weckhuysen, Mechanisms in Homogeneous and Heterogeneous Epoxidation Catalysis, Elsevier, Amsterdam, 2008, pp. 339–354.

[30] Y. Lu, W. Chen, Chem. Soc. Rev. 2012, 41, 3594–3623. 22441327 10.1039/c2cs15325d

[31] J. Lu, X. Zhang, J. J. Bravo-Suárez, K. K. Bando, T. Fujitani, S. T. Oyama, J. Catal. 2007, 250, 350–359.

[32a] J. Chen, S. J. A. Halin, E. A. Pidko, M. W. G. M. T. Verhoeven, D. M. P. Ferrandez, E. J. M. Hensen, J. C. Schouten, T. A. Nijhuis, ChemCatChem 2013, 5, 467–478;

[32b] X. Feng, X. Duan, J. Yang, G. Qian, X. Zhou, D. Chen, W. Yuan, Chem. Eng. J. 2015, 278, 234–239.

[33a] X. Feng, Z. Song, Y. Liu, X. Chen, X. Jin, W. Yan, C. Yang, J. Luo, X. Zhou, D. Chen, ACS Catal. 2018, 8, 10649–10657;

[33b] B. C. Bukowski, W. N. Delgass, J. Greeley, J. Phys. Chem. C 2021, 125, 4519–4531;

[34a] B. Chowdhury, J. J. Bravo-Suárez, M. Daté, S. Tsubota, M. Haruta, Angew. Chem. Int. Ed. 2006, 45, 412–415; 10.1002/anie.200502382 16342220

[34b] J. K. Edwards, A. F. Carley, A. A. Herzing, C. J. Kiely, G. J. Hutchings, Faraday Discuss. 2008, 138, 225–239; 18447018 10.1039/b705915a

[34c] M. Sankar, N. Dimitratos, P. J. Miedziak, P. P. Wells, C. J. Kiely, G. J. Hutchings, Chem. Soc. Rev. 2012, 41, 8099–8139; 23093051 10.1039/c2cs35296f

[34d] X. Feng, J. Yang, X. Duan, Y. Cao, B. Chen, W. Chen, D. Lin, G. Qian, D. Chen, C. Yang, X. Zhou, ACS Catal. 2018, 8, 7799–7808;

[34e] Z. Li, W. Ma, Q. Zhong, Ind. Eng. Chem. Res. 2019, 58, 4010–4016;

[34f] Z. Li, Z. Su, W. Ma, Q. Zhong, Appl. Catal. A 2021, 615, 118060;

[34g] L. Wang, J. Dai, Y. Xu, Y. Hong, J. Huang, D. Sun, Q. Li, J. Mater. Chem. A 2020, 8, 4428–4436;

[34h] J. Huang, T. Takei, T. Akita, H. Ohashi, M. Haruta, Appl. Catal. B 2010, 95, 430–438;

[34i] Y.-g. Ren, J. Huang, Q. Lv, Y. Xie, A.-H. Lu, M. Haruta, Appl. Catal. A 2019, 584, 117172;

[34j] Z. Song, X. Feng, N. Sheng, D. Lin, Y. Li, Y. Liu, X. Chen, D. Chen, X. Zhou, C. Yang, Chem. Eng. J. 2019, 377, 119927.

[35a] A. K. Sinha, S. Seelan, S. Tsubota, M. Haruta, Angew. Chem. Int. Ed. 2004, 43, 1546–1548; 10.1002/anie.200352900 15022229

[35b] C. Qi, M. Okumura, T. Akita, M. Haruta, Appl. Catal. A 2004, 263, 19–26.

[36] D. P. Anderson, R. H. Adnan, J. F. Alvino, O. Shipper, B. Donoeva, J.-Y. Ruzicka, H. Al Qahtani, H. H. Harris, B. Cowie, J. B. Aitken, Phys. Chem. Chem. Phys. 2013, 15, 14806–14813. 23907108 10.1039/c3cp52497c

[37] B. G. Donoeva, D. S. Ovoshchnikov, V. B. Golovko, ACS Catal. 2013, 3, 2986–2991.

[38] Y. Yuan, K. Asakura, A. P. Kozlova, H. Wan, K. Tsai, Y. Iwasawa, Catal. Today 1998, 44, 333–342.

[39] X. Duan, X. Tian, J. Ke, Y. Yin, J. Zheng, J. Chen, Z. Cao, Z. Xie, Y. Yuan, Chem. Sci. 2016, 7, 3181–3187. 29997810 10.1039/c5sc04283f PMC6005270

[40] B. Yoon, H. Häkkinen, U. Landman, A. S. Wörz, J.-M. Antonietti, S. Abbet, K. Judai, U. Heiz, Science 2005, 307, 403–407. 15662008 10.1126/science.1104168

[41] Z. Li, Y. Wang, J. Zhang, D. Wang, W. Ma, Catal. Commun. 2017, 90, 87–90.

[42] H. Hosseiniamoli, A. Setiawan, A. A. Adesina, E. M. Kennedy, M. Stockenhuber, Catal. Sci. Technol. 2020, 10, 1193–1204.

[43] A. M. Joshi, W. N. Delgass, K. T. Thomson, J. Phys. Chem. B 2006, 110, 16439–16451. 16913775 10.1021/jp061754o

[44] B. K. Min, W. Wallace, D. Goodman, J. Phys. Chem. B 2004, 108, 14609–14615.

[45] R. Sharma, G. P. Holland, V. C. Solomon, H. Zimmermann, S. Schiffenhaus, S. A. Amin, D. A. Buttry, J. L. Yarger, J. Phys. Chem. C 2009, 113, 16387–16393.

[46] Y. Wang, J. Zhuang, G. Yang, D. Zhou, D. Ma, X. Han, X. Bao, J. Phys. Chem. B 2004, 108, 1386–1391.

[47] W. Wang, R. W. Murray, Langmuir 2005, 21, 7015–7022. 16008417 10.1021/la0508700

[48] P. Zhao, B. Boekfa, T. Nishitoba, N. Tsunoji, T. Sano, T. Yokoi, M. Ogura, M. Ehara, Microporous Mesoporous Mater. 2020, 294, 109908.

[49] E. Duprey, J. Maquet, P. Man, J.-M. Manoli, M. Delamar, J.-M. Brégeault, Appl. Catal. A 1995, 128, 89–96.

