# Supplementary material for: Precisely Engineered Supported Gold Clusters as a Stable Catalyst for Propylene Epoxidation
Source: Angew Chem Int Ed Engl. 2021 Jul 9;60(33):18185–93. doi: 10.1002/anie.202104952 (PMC8456944; doi:10.1002/anie.202104952)
Supplement: Supplementary file 1 — Supporting Information [file ANIE-60-18185-s001.pdf]

Angewandte Chemie
Eine Zeitschrift der Gesellschaft Deutscher Chemiker

Supporting Information

Precisely Engineered Supported Gold Clusters as a Stable Catalyst for Propylene Epoxidation

Nidhi Kapil, Tobias Weissenberger, Fabio Cardinale, Panagiotis Trogadas, T. Alexander Nijhuis, Michael M. Nigra, and Marc-Olivier Coppens**

anie_202104952_sm_miscellaneous_information.pdf

Table of Contents

Materials and methods 1
Supplementary figures...4
References 10

Materials and methods

Chemicals:
Ethanol (Merck), methanol (Sigma Aldrich), hexane (Sigma Aldrich), gold(III) chloride trihydrate (Sigma Aldrich), triphenylphosphine (Sigma Aldrich), sodium borohydride (Sigma Aldrich), ammonia solution (Sigma Aldrich), tetraethyl orthosilicate (Sigma Aldrich), tetra-propyl ammonium hydroxide (Merck), tetra-butyl orthotitanate (Merck), lithium tetraborate (Sigma Aldrich), nitric acid (Sigma Aldrich), hydrochloric acid (Sigma Aldrich), Poly(tetrafluoroethylene) (Sigma Aldrich), silicon carbide (Alfa Aesar) were received and used as it is. Ultra-high purity (UHP) grade helium, nitrogen and oxygen cylinders were procured from BOC Ltd and propylene from Air Liquide.

Synthesis of phosphine bound gold clusters:
Sub-nanometre gold clusters were synthesised using a one-pot methodology. First, 4.5 mg of gold precursor ($HAuCl_4 \cdot 3H_2O$) was dissolved in ~34 mL of ethanol in a 100mL round bottom flask. After 15 min of magnetic stirring, an ethanolic solution of 10 eq. of triphenylphosphine w.r.t. Au atoms was added; this was followed by the addition of finely ground sodium borohydride ($NaBH_4$). The concentration of reducing agent was optimised by varying the amount of $HAuCl_4$/$NaBH_4$ (2, 4, 10, 20 equivalents per Au atom in the preparation of each solutions). Au clusters were found to be stable with 4 eq. of $NaBH_4$. This mixture was further stirred for 2h to obtain a homogeneous dispersed solution, all at room temperature. The colour of the solution changed from yellow to clear and, eventually, to orange.

Synthesis of titanium silicalite-1 (TS-1) support:
TS-1 was synthesised using a reported protocol.[1] Briefly, 23 g of tetraethyl orthosilicate (TEOS) and 12.8 g of tetra-propyl ammonium hydroxide (TPAOH, 40 wt. % aqueous solution) were mixed and stirred in a polyethene beaker. 1.1 g of tetra-butyl orthotitanate (TBOT) were separately dissolved in 6.3 mL of isopropanol. This solution was then added dropwise for 15 minutes to the TEOS mixture to obtain a clear solution under continuous stirring. This was followed by the addition of another 4.7 g of TPAOH. The final mixture was heated at 80 ºC for 3h to remove isopropanol. After the addition of 35 mL of water, the resulting gel was placed in PTFE-lined autoclave (Parr Instruments) for crystallisation at 170 ºC for 24h. The product was separated by centrifugation, washed three times with 20-25 mL of deionised water, and dried overnight in air at 80 ºC. After drying, the white powder was calcined at 550 ºC for 5h (heating at 1ºC/min).

Catalyst preparation procedures

Colloidal immobilisation

Sub-nanometre clusters synthesised by the one pot methodology were used for the immobilisation onto the supports. 1 g of the TS-1 powder was placed in the round bottom flask. 50 mL of Au clusters in ethanol were poured onto the supports to obtain 1 wt % final Au loading. Ethanol was removed using a rotatory evaporator at 45 ˚C under vacuum. After the removal of the solvent, orange powder was obtained, and it was dried overnight before purification. The dried solid powder was dispersed in hexane and washed 3 times using a water and hexane (1:1) mixture. The ligand was removed using a non-thermal plasma at room temperature. The catalyst powder was placed in a plasma chamber (Henniker HPT-200) operated at 750 VA and 0.5 mbar pressure for 30 minutes. The treatment was performed in 3 cycles of 10 minutes each. After each cycle, the powder was mixed to facilitate uniform removal of ligand and dissipate any heat from the sample.

Calcination in air

The catalyst powder was calcined under air in a chamber furnace (Carbolite) at a temperature of 300˚C. The temperature was increased at a rate of 5 ˚C/min to the final temperature; this was maintained for 4h before cooling back to room temperature.

Deposition precipitation method

Gold was also deposited onto TS-1 by using the deposition precipitation method.[1] In a typical synthesis, 2 g of TS-1 powder was dispersed in 100 mL of deionised water. The pH of the dispersion was raised to 9-10 by adding dropwise a solution of ammonia (2.5 wt.%). Approximately, 40 mg of gold precursor ($HAuCl_4.3H_2O$) was dissolved in 20 mL of deionised water to obtain the nominal gold loading of 1 wt.%. The gold solution was added dropwise to the above mixture in 15 minutes and then the slurry was allowed to stir vigorously for 1 h. While stirring, the pH of the catalyst slurry was monitored and maintained at ~9.5 to facilitate precipitation of gold. The solid was centrifuged and washed three times by suspending the catalyst powder in deionised water. Finally, the catalyst was dried overnight at 80 ºC, followed by calcination at 400 ºC (heating at 5 ºC/min). This procedure was very light sensitive, so that amber glassware was used for the entire synthesis, and the synthesis was performed in the dark. The catalyst was also stored in amber vials.

Catalyst characterisation

UV/Vis for colloids: UV/Vis absorption spectra were collected at room temperature using a Cary 4000 UV/Vis spectrophotometer using a quartz cuvette. The ethanolic gold solution was subjected to spectroscopy immediately after the synthesis. All the data were corrected using the same solvent for background absorptions.

UV/Vis for powder: Diffuse reflectance UV/Vis spectra of the supported powder were recorded by using a Cary 4000 UV/Vis spectrophotometer attached to a praying mantis (solid sample cell attachment, Harrick Scientific), using Teflon as a reference.

Electron microscopy: Transmission electron microscopy (TEM) images were obtained using a JEOL 2100 instrument operating at 200 kV. The samples were dispersed in ethanol. Then, the dispersion was drop-casted to holey carbon-coated copper grids (EM resolutions Ltd) and dried before imaging under the microscope. The particle size analysis was performed using ImageJ software.

Powder XRD: XRD patterns of the powder were collected on a Stoe STADI-P diffractometer with Cu Kα radiation (λ = 0.15406 nm) working at 40 kV and 30 mA.

Nitrogen physisorption: Nitrogen adsorption and desorption isotherms of the samples were measured using a Quantachrome Autosorb iQ2 automated gas sorption analyser. Samples were outgassed at 380ºC for 8 h before analysis. The specific surface areas of the samples were estimated using the standard Brunauer-Emmett-Teller (BET) method, and their pore size distributions were calculated using Non-Local Density Functional Theory (NLDFT).[1]

TGA: Thermal gravimetric analysis of the catalyst powders was performed in air, using a Mettler Toledo TGA/DSC3+. The temperature was increased at a rate of 10 °C/min from room temperature to 800 °C.

ICP-OES: The final gold loading was calculated using Varian 720 inductive coupled plasma optical emission spectrometry (ICP-OES). Sample preparation was done by flux melting using lithium tetraborate, followed by dissolution in nitric acid and aqua regia.[2]

XPS: The powder samples of untreated and plasma treated Au/TS-1 were dried in vacuum at 60 °C overnight, prior to X-ray photoelectron spectroscopy (XPS) measurements. A survey scan between 0 and 1200 eV was performed for each sample. Then, high-resolution scans (100 per element) for gold and phosphorus were conducted with 20 eV pass energy. CasaXPS software was used for data processing. The peak area ratio of the Au 4f doublet ($f_{5/2}$:$f_{7/2}$) was fixed to 3:4.

^{31}P MAS NMR: Solid-state NMR experiments were carried out on Bruker Advance 300 spectrometer with 7.05 T wide-bore magnet at ambient probe temperature. High-resolution solid-state ^{31}P NMR spectra were recorded at 121.5 MHz using a standard Bruker 4 mm double-resonance magic-angle spinning (MAS) probe and high-power proton decoupling. Solid materials were packed into zirconia rotors of 4 mm external diameter and spun at the MAS frequency of 12 kHz with a stability better than ±3 Hz. Typical acquisition

conditions for ^{31}P MAS experiments were: ^{29}Si 30° pulse duration = 1.5 µs; recycle delay = 600 s; acquisition time = 45 ms. ^{31}P chemical shifts are given relative to an 85% aqueous solution of H_3PO_4 (0 ppm).

Catalytic testing

The direct gas phase epoxidation of propylene was carried out in a fixed packed bed reactor. 0.5 g of catalyst powder was diluted with 2.5 g of silicon carbide (SiC/catalyst weight ratio = 5) to dissipate heat within the catalyst bed. The catalyst was loaded into the quartz reaction tube (outer diameter 9 mm, wall thickness 1.5 mm) placed inside a tubular furnace (Carbolite Gero) and equipped with a fixed K-type probe thermocouple to measure the reaction temperature. The catalytic performance was measured at a reaction temperature of 200 ºC. The reaction feed consisted of 10/10/10/70 vol% of $C3H_6/H_2/O_2$/He, respectively, with a total flowrate of 66.67 mL min^{-1}, resulting in a gas hourly space velocity (GHSV) of 8000 mL $gcat^{-1}$ h^{-1}. The concentrations of reactants and products were obtained by online gas chromatography (GC, Shimadzu). The oxygenates (PO, ethanal, propanal, acetone and acrolein), along with CO_2, H_2O and C_3H_6, were separated using a Porapak T column and analysed by a flame ionisation detector (FID), while the permanent gases (H_2, O_2, CO) were separated by a Molsieve 5A column and analysed by a thermal conductivity detector (TCD). A blank experiment was performed, which confirmed that no PO was generated in the absence of catalyst. The C_3H_6 conversion, PO formation rate, PO selectivity and H_2 efficiency were calculated as follows:[3]

$$C_3H_6 \text{ conversion } (\%) = \frac{\frac{1}{3}F_{COx}^{out} + \frac{2}{3}F_{ethanal}^{out} + \sum F_{C3_{oxy}}^{out}}{F_{C3H6}^{in}} * 100$$

$$\text{PO formation rate } (g_{PO}\ h^{-1}\ kg_{cat}^{-1}) = \frac{F_{PO}^{out}}{\text{catalyst weight}} * M_{PO} * 60$$

$$\text{PO selectivity } (\%) = \frac{F_{PO}^{out}}{\frac{1}{3}F_{COx}^{out} + \frac{2}{3}F_{ethanal}^{out} + \sum F_{C3_{oxy}}^{out}} * 100$$

$$H_2 \text{ efficiency } (\%) = \frac{F_{PO}^{out}}{F_{H2}^{in} - F_{H2}^{out}} * 100$$

$$H_2 \text{ conversion } (\%) = \frac{F_{H2}^{in} - F_{H2}^{out}}{F_{H2}^{in}} * 100$$

$$O_2 \text{ conversion } (\%) = \frac{F_{O2}^{in} - F_{O2}^{out}}{F_{O2}^{in}} * 100$$

Where F_i is the molar flow rate of the generic i^{th} species, in mol min^{-1}, catalyst weight is in kg and M_{PO} is the molar mass of propylene oxide in g/mol.

Supplementary figures

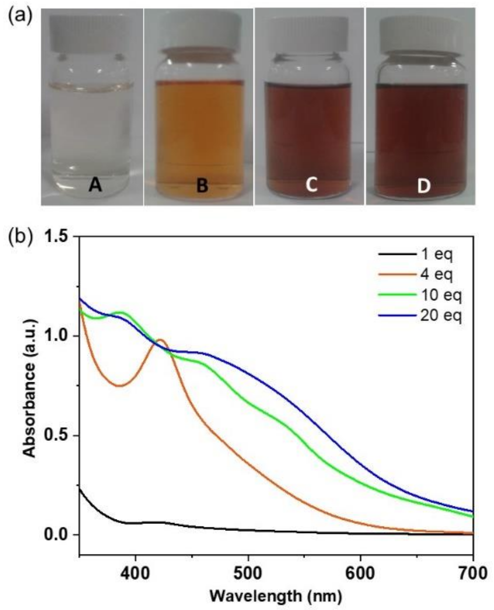

Figure S1 **Optimisation of Au nanoclusters.** a. Glass vials showing Au nanoparticle solutions in ethanol synthesised using different amounts of sodium borohydride (A: 1 eq.; B: 4 eq.; C: 10 eq.; D: 20 eq.). b. Corresponding UV/Vis spectra of the gold nanoparticle solutions.

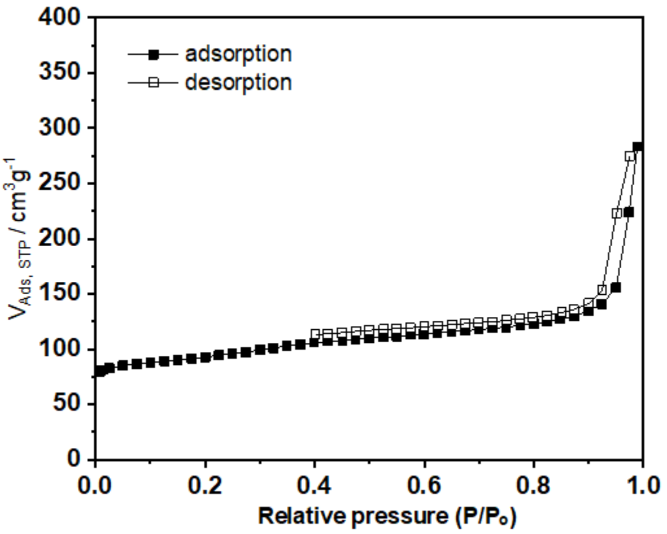

Sample	Surface Area (m^2/g)	Micropore Vol. (cm^3/g)
TS-1	548	0.17

Figure S2 **N_2 adsorption and desorption isotherms** of powdered TS-1, BET surface area and micropore volume.

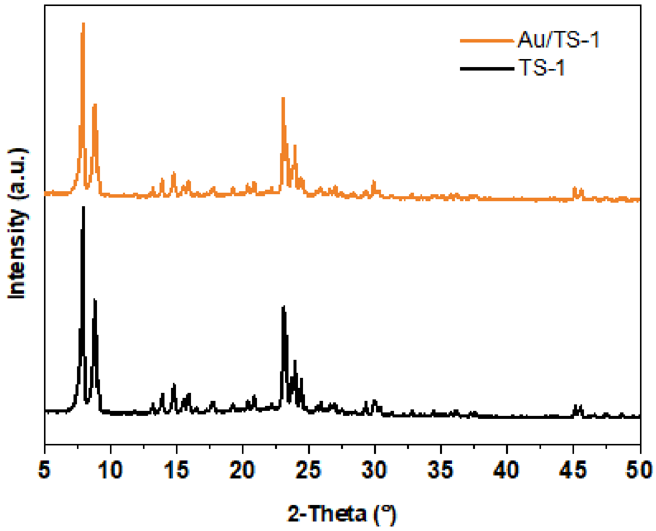

Figure S3 **Powder X-ray diffraction (XRD) spectra** of TS-1 and Au/TS-1.

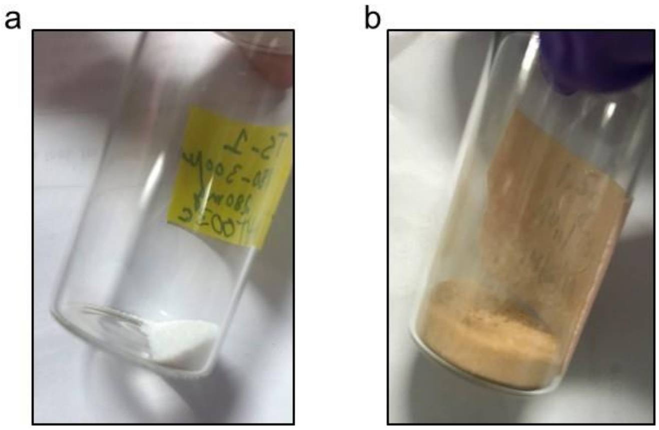

Figure S4 **Photographs of the vial with sample.** a) TS-1. b) Au/TS-1.

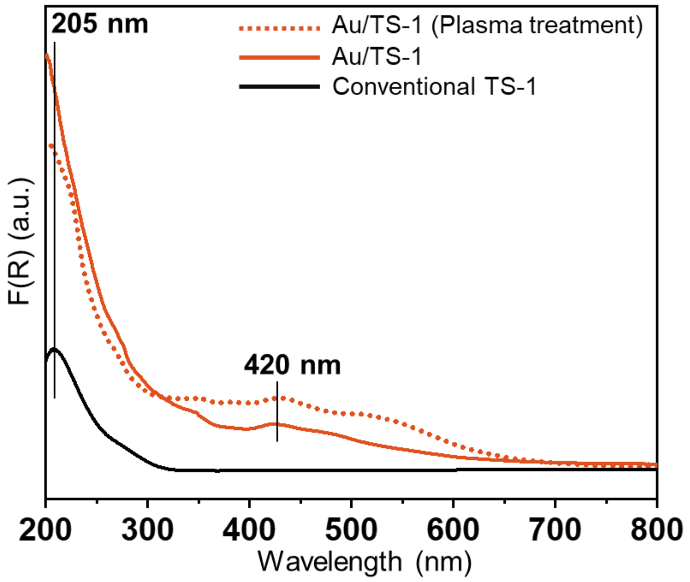

Figure S5 **DR-UV/Vis spectroscopy.** TS-1, Au/TS-1 and Au/TS-1PT catalyst.

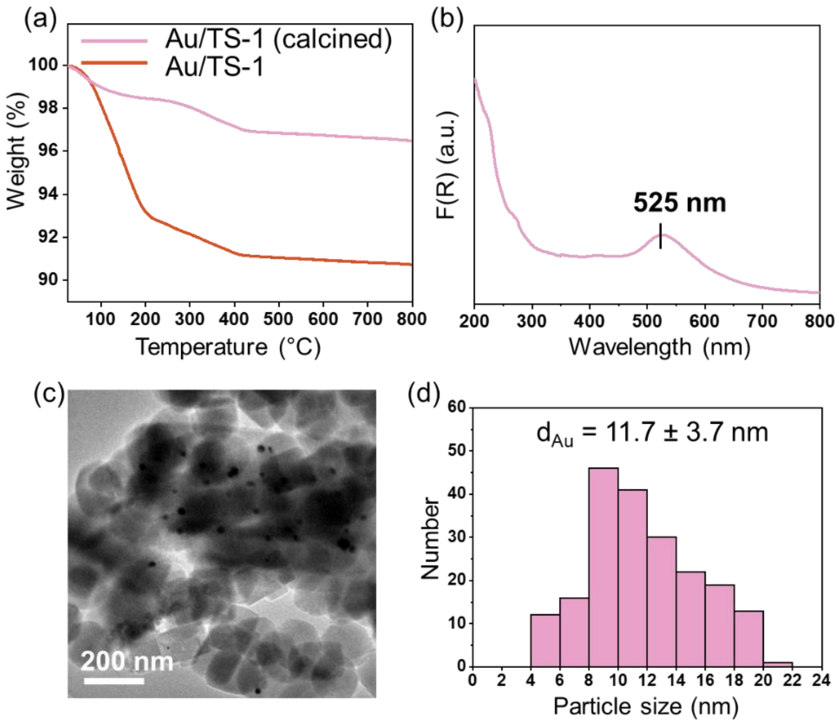

Figure S6 **Characterisation of calcined Au/TS-1 catalyst.** a) TGA. b) DR-UV/VIS spectrum. c)TEM. d) Particle size distribution histogram.

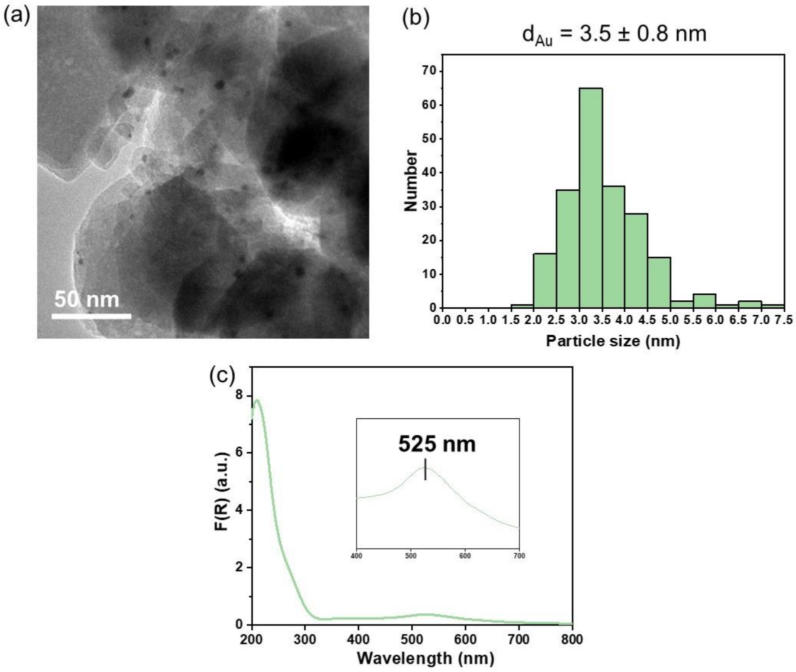

Figure S7 **Characterisation of the untreated Au/TS-1$_{DP}$ catalyst.** a) TEM. b) Particle size distribution histogram. c) DR-UV/VIS spectrum; inlay shows the spectrum between 400 to 700 nm.

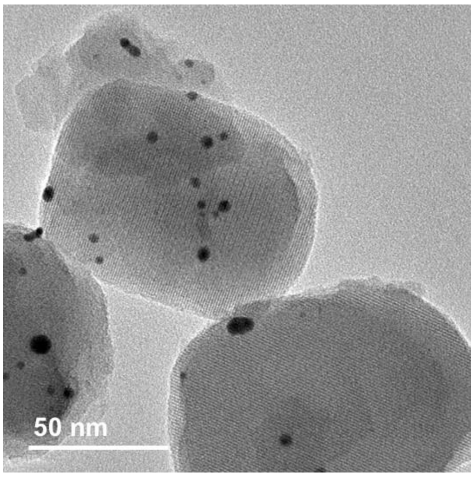

Figure S8 **HRTEM micrograph.** Au/TS-1$_{PT}$ spent catalyst.

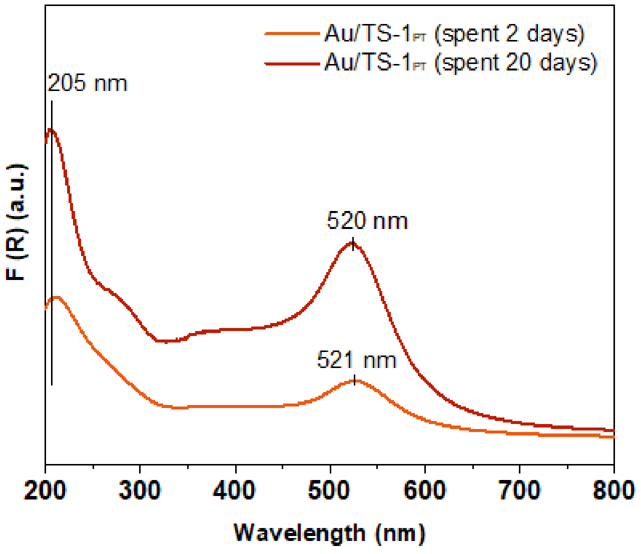

Figure S9 **DR-UV/Vis Spectrum of Au/TS-1$_{PT}$ catalyst.** Spectra collected after 2 and 20 days of reaction time on stream.

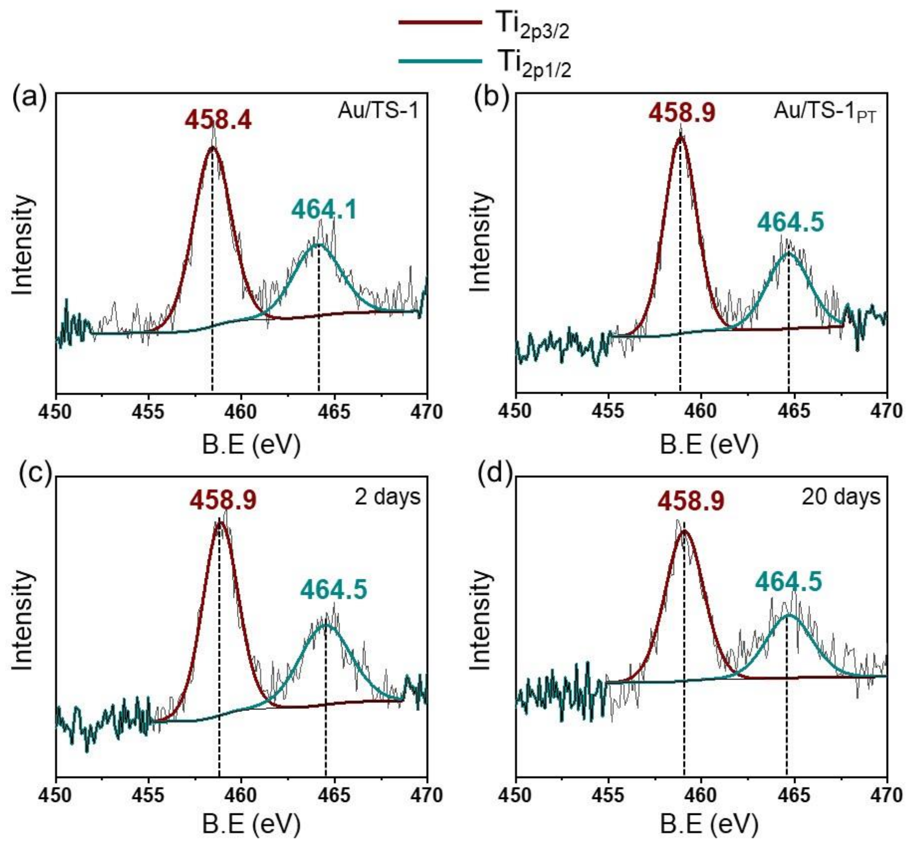

Figure S10 **XPS spectra of titanium for Au/TS-1 catalyst.** a) as synthesised. b) plasma treated (PT). c) after 2 days of reaction. d) after 20 days of reaction.

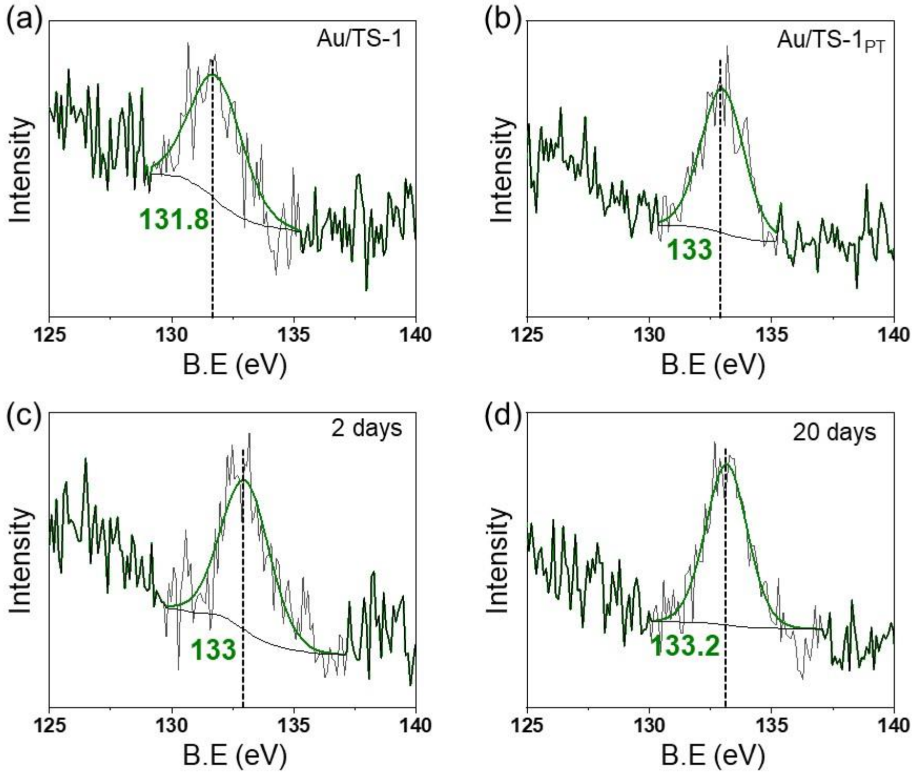

Figure S11 **XPS spectra of phosphorus of Au/TS-1 catalyst.** a) as synthesised. b) plasma treated (PT). c) after 2 days of reaction. d) after 20 days of reaction.

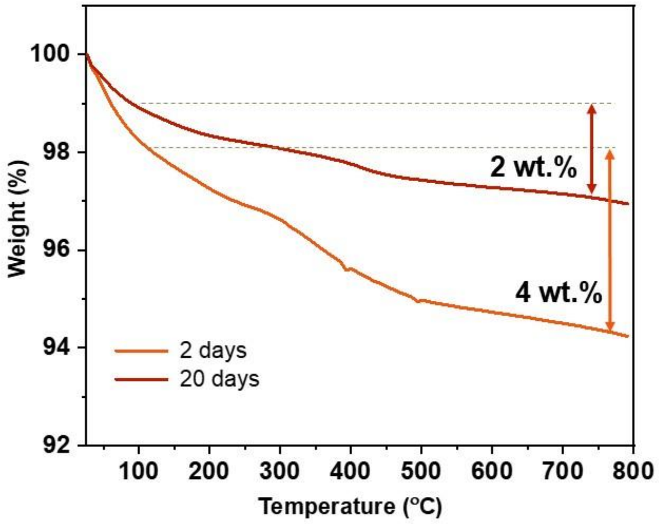

Figure S12 **TGA analysis of Au/TS-1$_{PT}$ catalyst.** Spectra collected after 2 and 20 days of reaction time on stream.

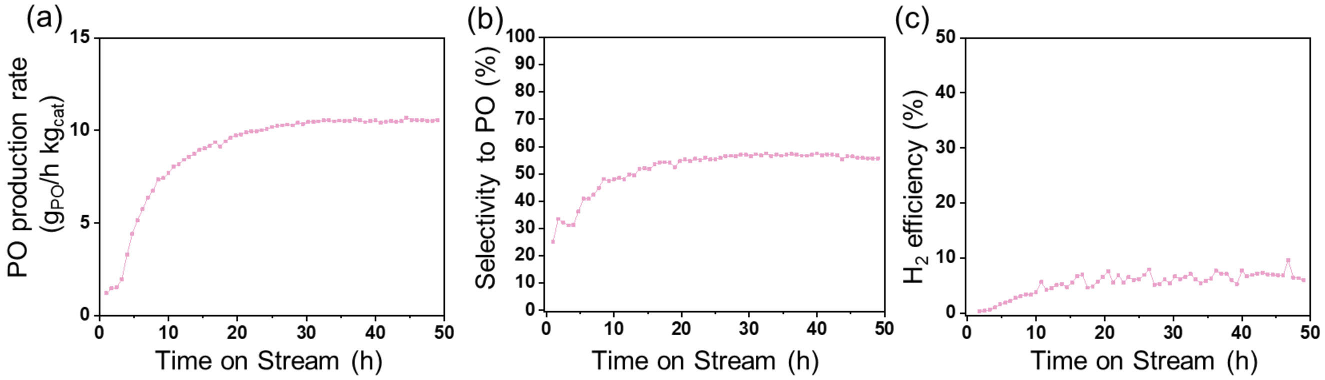

Figure S13 **Catalytic performance of calcined Au/TS-1 catalysts** a) PO production rate. b) PO selectivity. c) Hydrogen efficiency.

References

[1] P. I. Ravikovitch, A. V. Neimark, *The Journal of Physical Chemistry B* **2001**, *105*, 6817-6823.
[2] A. Wittmann, F. Kop, *Spectrochimica Acta Part B: Atomic Spectroscopy* **1986**, *41*, 73-79.
[3] W.-S. Lee, M. Cem Akatay, E. A. Stach, F. H. Ribeiro, W. Nicholas Delgass, *J. Catal.* **2012**, *287*, 178-189
